# Deletion of sRNA0024 Reduces Virulence of *Pseudomonas plecoglossicida* and Alleviates Host Immune Injury in *Epinephelus coioides*

**DOI:** 10.3390/ani15243623

**Published:** 2025-12-17

**Authors:** Lingmin Zhao, Yihai Ouyang, Jiang Zheng, Yujia Sun, Yingxue Qin, Meiqin Mao

**Affiliations:** Fisheries College, Jimei University, Xiamen 361021, China; ouyang021225@outlook.com (Y.O.); zhengjiang618@163.com (J.Z.); sunyj@jmu.edu.cn (Y.S.); yxqin@jmu.edu.cn (Y.Q.); meiqinmao0623@gmail.com (M.M.)

**Keywords:** *luxR*, sRNA, post-transcriptional regulation, *Epinephelus coioides*, *Pseudomonas plecoglossicida*, antivirulence target, host immunity

## Abstract

Visceral white spot disease caused by *Pseudomonas plecoglossicida* severely threatens marine aquaculture, highlighting the need for effective control strategies. To clarify the role of a novel small RNA, sRNA0024, in bacterial pathogenicity, we constructed an sRNA0024 deletion mutant (ΔsRNA0024) and compared its phenotype and virulence with those of the wild-type strain NZBD9. In vitro assays showed that deletion of sRNA0024 did not affect bacterial growth but significantly reduced biofilm formation and adhesion. In vivo infection experiments in orange-spotted grouper (*Epinephelus coioides*) demonstrated that the ΔsRNA0024 mutant had a 3.8-fold higher 50% lethal dose (LD_50_), improved host survival, and milder splenic lesions than the wild type. Histopathology and host transcriptome analyses revealed weakened activation of complement–coagulation cascades, neutrophil extracellular traps, leukocyte migration, and inflammatory signaling pathways, indicating a lower-intensity immune response. Bacterial transcriptomics showed that deletion of sRNA0024 was associated with reduced *luxR* expression and attenuated quorum-sensing–associated virulence traits, supporting a possible role for this small RNA in modulating *luxR* expression and QS-related host immunopathology. These findings identify sRNA0024 as an important contributor to the virulence of *P. plecoglossicida* and highlight the sRNA0024–*luxR* module as a potential antivirulence target for controlling visceral white spot disease in aquaculture.

## Simple Summary

Visceral white spot disease caused by the bacterium *Pseudomonas plecoglossicida* leads to high mortality in farmed groupers and other marine fish, creating serious economic losses for aquaculture. To explore new ways to control this disease, we focused on sRNA0024, a small regulatory molecule in this bacterium that can influence the activity of many genes. We removed sRNA0024 from a highly virulent strain and compared the altered strain with the original one using laboratory tests and infection experiments in orange-spotted grouper (*Epinephelus coioides*). The strain lacking sRNA0024 formed weaker biofilms, attached less effectively to host tissues, and caused fewer deaths and milder spleen damage in infected fish. Further analyses showed reduced activity of bacterial genes involved in cell-to-cell communication and weaker activation of fish genes related to inflammation and tissue injury. These results suggest that targeting the sRNA0024–*luxR* regulatory pathway may help develop new antivirulence approaches to protect farmed fish from visceral white spot disease.

## 1. Introduction

*Pseudomonas plecoglossicida* is a Gram-negative, short rod-shaped bacterium widely distributed in seawater, freshwater, and soils, and it poses a major threat to the sustainable aquaculture of freshwater and marine fish species [1]. It is an aerobic, non–spore-forming, and noncapsulated organism [2]; on Luria–Bertani (LB) agar, it forms smooth-edged, circular, and semi-translucent yellowish-white colonies [3]. Recent studies have established *P. plecoglossicida* as the primary etiological agent of visceral white spot disease in farmed large yellow croaker (*Larimichthys crocea*) in Fujian and Zhejiang provinces, China [4,5,6]. Experimental infections further demonstrated that *P. plecoglossicida* can induce characteristic visceral white spot lesions in orange-spotted grouper (*Epinephelus coioides*) [7], *Nibea albiflora* [8], and *Lates calcarifer* [9]. In the early stages of infection, affected fish often exhibit lethargy and inappetence with few external signs and low mortality; as the disease progresses, mass mortality may occur, and necropsy typically reveals variably sized white nodules distributed on the spleen, liver, and kidney [2,10]. Numerous studies have also explored prevention and therapeutic measures against *P. plecoglossicida*-associated visceral white spot disease [11,12,13]. Furthermore, genomic and comparative analyses indicate that its virulence involves multilayered regulatory networks and complex host–pathogen interactions encompassing adhesion, invasion, metabolic reprogramming, and immune evasion [1,14,15,16,17]. Therefore, identifying upstream regulatory nodes is essential for elucidating the pathogenic mechanisms and developing anti-virulence strategies.

The *luxR* gene encodes a transcriptional regulator that plays a pivotal role in bacterial quorum sensing (QS). First identified in *Vibrio fischeri*, *luxR* controls the bacterial bioluminescence system [18]. The LuxR protein functions as a central transcription factor within the QS regulatory network and modulates diverse biological processes, including light production, virulence factor secretion, and biofilm formation [19]. In our previous study, RNA interference (RNAi) was employed to silence *luxR* in *P. plecoglossicida*. The pathogenicity of the *luxR*-RNAi strain was compared with that of the wild type, and the host immune responses were analyzed in artificially infected striped grouper. The *luxR*-silenced mutant exhibited an approximately 30% reduction in mortality compared with the wild-type strain. Transcriptomic profiling further revealed that infection with the *luxR*-RNAi strain induced marked alterations in the grouper’s immune defense, with significant transcriptional changes concentrated in the Nod-like receptor (NLR) signaling pathway. These findings demonstrate that *luxR* is a critical determinant of host–pathogen interactions: reduced *luxR* expression weakens QS signaling while enhancing *IL-1β* expression within the host NLR pathway. This likely triggers a compensatory proinflammatory response, thereby strengthening the host’s immune defense against infection [20].

Small RNAs (sRNAs) are non-coding RNA molecules, typically 50–500 nucleotides in length, which play widespread roles in regulating bacterial gene expression [21,22,23]. By sensing environmental changes and acting at the post-transcriptional level, sRNAs modulate the expression of target genes and thereby influence bacterial virulence and host immune responses [24,25,26]. Post-transcriptional regulation by sRNAs represents an efficient and economical strategy that enables pathogens to adapt to host environments and fine-tune virulence gene expression [27,28,29]. In recent years, sRNAs have been recognized as key mediators of bacterial adaptation to environmental stress and as crucial determinants of virulence-associated phenotypes. Classic studies demonstrated that the LuxR protein functions as a positive regulator by binding acyl-homoserine lactone (AHL) signals to activate downstream gene expression [30,31]. Subsequent work revealed that the Qrr sRNA family base-pairs with *luxR* mRNA, promoting its degradation or blocking its translation—thereby uncovering an indirect sRNA-mediated mechanism controlling *luxR* [32,33,34]. This interaction typically depends on the RNA chaperone Hfq, which facilitates and stabilizes sRNA–mRNA pairing [35,36]. Nevertheless, major gaps remain: (i) the complexity of sRNA regulatory networks—particularly their interactions with other regulators—has not been fully elucidated; (ii) sRNA functions are often species-specific, and many remain undiscovered; (iii) functional analyses have relied mainly on in vitro systems, with limited validation under host-like conditions; and (iv) the temporal dynamics of sRNA regulation remain poorly characterized. Further elucidation of the regulatory relationship between sRNAs and *luxR* will not only advance understanding of quorum-sensing circuitry and bacterial behavioral control but also provide promising targets for the development of antivirulence and antimicrobial strategies.

In *Pseudomonas* species, several small RNAs have been shown to modulate quorum sensing, biofilm formation and virulence by targeting global regulators and signaling components, underscoring the importance of post-transcriptional control in pathogen adaptation. For example, the sRNAs PhrS, AmiL, PrrF1/2, ReaL and RsmY/RsmZ/RsmW in *Pseudomonas aeruginosa* participate in the regulation of quorum sensing networks, biofilm development and multiple virulence factors [37,38,39,40,41]. However, sRNA-mediated regulation of *luxR*-type transcriptional regulators in *P. plecoglossicida* has not yet been explored, and the contribution of such sRNAs to visceral white spot disease remains unclear.

This study aimed to elucidate the impact of the *luxR* gene on *P. plecoglossicida*, with particular emphasis on the role of small RNAs (sRNAs) in its post-transcriptional regulation. We computationally predicted sRNAs targeting *luxR* and constructed a *P. plecoglossicida* mutant lacking the candidate sRNA, systematically examining its effects on *luxR* expression and on downstream regulatory pathways, phenotypes, and functions. Using in vitro bacterial assays and a host infection model, we validated the regulatory influence of this sRNA on virulence factor secretion and host immune responses. At the systems level, this work clarifies the regulatory logic underlying *P. plecoglossicida* pathogenesis and, from an antivirulence perspective, provides a theoretical foundation and potential molecular targets for controlling visceral white spot disease in large yellow croaker (*Larimichthys crocea*).

## 2. Materials and Methods

### 2.1. Bacterial Strains, Plasmid Construction and Cultivation Conditions, and Experimental Fish

The highly pathogenic *P. plecoglossicida* strain NZBD9 was isolated from the spleen of *Larimichthys crocea* exhibiting visceral white spot disease [2]. *E. coli* strains DH5α and TOP10 (Tsingke Biotech, Beijing, China) were used for plasmid propagation and mutant construction. *P. plecoglossicida* and *E. coli* were cultured at 18 °C and 37 °C, respectively, with shaking at 220 rpm in Luria–Bertani (LB) broth or on LB agar plates (1.5% agar; Guangdong Huankai Microbial Sci. & Tech. Co., Ltd., Guangzhou, China). When necessary, the medium was supplemented with antibiotics: ampicillin (Amp, 50 μg/mL), kanamycin (Kan, 50 μg/mL), or tetracycline (Tet, 10 μg/mL) (all from Xilong Scientific Co., Ltd., Shantou, China). Amp was used for routine culture of *P. plecoglossicida*, Kan for selection of deletion mutants, and Tet for selection of complemented strains.

Healthy *E. coioides* (♀ × ♂; approximately 14 cm in total length and 50 g in body weight) were obtained from a marine aquaculture farm in Zhangpu, Fujian Province, China. The fish were acclimated for two weeks in an indoor recirculating seawater system before experimental infection. During acclimation, continuous aeration was provided, the water temperature was maintained at 18 ± 1 °C, and the fish were fed once daily with a commercial grouper diet.

### 2.2. Prediction of sRNAs in P. plecoglossicida

To investigate how sRNAs regulate *luxR*, we first used the prokaryotic RNA-seq analysis software Rockhopper (version 2.03; http://cs.wellesley.edu/~btjaden/Rockhopper/ (accessed on 15 June 2025)) to identify candidate sRNAs [42]. The putative targets of these sRNAs were subsequently predicted using RNAhybrid (version 2.1.2; https://bibiserv.cebitec.uni-bielefeld.de/rnahybrid (accessed on 15 June 2025)) and RNAplex (version 0.2; http://legacy.bioinf.uni-leipzig.de/Software/RNAplex/ (accessed on 15 June 2025)) [43]. Potential base-pairing interactions between sRNA0024 and *luxR* mRNA were analyzed with IntaRNA (version 3.4.1; https://rna.informatik.uni-freiburg.de/IntaRNA/Input.jsp (accessed on 15 June 2025)) [44], and the secondary structure of sRNA0024 was predicted using RNAfold (ViennaRNA Package version 2.7.0; http://rna.tbi.univie.ac.at/cgi-bin/RNAWebSuite/RNAfold.cgi (accessed on 15 June 2025)) [45].

### 2.3. Construction of the ΔsRNA0024 Mutant in P. plecoglossicida

Based on bioinformatic predictions, we identified a small RNA putatively targeting *luxR* mRNA, designated sRNA0024; its nucleotide sequence is presented in Results Section 3.1. Using the sRNA0024 locus as reference, two primer pairs (UA-F/UA-R and DA-F/DA-R) were designed to amplify the upstream (330 bp) and downstream (656 bp) homologous arms for allelic exchange. The primer sequences were as follows: sRNA0024-up-F, 5′-TACGGAGCGGGTTGCGCGAG-3′; sRNA0024-up-R, 5′-GCGCTCCCGTCGCGCGGTGC-3′; sRNA0024-down-F, 5′-TGCTCACGCCGCAGGCTCTG-3′; and sRNA0024-down-R, 5′-CTGCAGGAAATCGTCCGGCC-3′. A two-step allelic exchange strategy was performed using the suicide plasmid pK18mobsacB [46]. Genomic DNA of *P. plecoglossicida* served as the PCR template, and the upstream and downstream homologous arms were amplified separately with primer pairs sRNA0024-up-F/R and sRNA0024-down-F/R, respectively. The PCR mixture (25 μL) contained 1.0 μL template DNA (105.2 μg/mL), 1.0 μL of each primer (10 μmol/L), 12.5 μL of 2× Pfu Master Mix (Beijing Lanbo Biotech Co., Ltd., Beijing, China), and nuclease-free water to a final volume of 25 μL. The PCR cycling conditions were as follows: initial denaturation at 94 °C for 3 min; 34 cycles of 94 °C for 30 s, 60 °C for 30 s, and 72 °C for 1 min; and a final extension at 72 °C for 5 min, yielding the expected upstream and downstream fragments of the sRNA0024 locus.

Subsequently, the upstream and downstream homologous arms were combined at a 1:1 molar ratio, and 1 μL of the mixture was used as the template for fusion PCR. The reaction was performed with primers sRNA0024-up-F and sRNA0024-down-R. The 50 μL PCR mixture contained 1 μL each of the upstream and downstream homologous arm fragments, 2.0 μL of each primer (10 μmol/L), 25 μL of 2× Pfu Master Mix (Beijing Lanbo Biotech Co., Ltd., Beijing, China), and nuclease-free water to a final volume of 50 μL. The amplification program consisted of an initial denaturation at 94 °C for 3 min, followed by 34 cycles of 94 °C for 30 s, 60 °C for 30 s, and 72 °C for 90 s, yielding the final fusion PCR product.

The pK18mobsacB vector was digested with restriction enzymes *EcoR I* and *Xba I* (Beijing Baori Yi Biotechnology Co., Ltd., Beijing, China) at 37 °C for 5 h to generate a linearized plasmid backbone. This step was part of a two-step allelic exchange procedure employing the suicide plasmid pK18mobsacB [47]. The purified fusion PCR fragment was then ligated into the digested vector using a seamless cloning kit (NuoWeizhan Biotechnology Co., Ltd., Shanghai, China, catalog no. C112) at 16 °C for 12 h, yielding the recombinant plasmid construct.

The recombinant plasmid was introduced into competent *P. plecoglossicida* cells by electroporation at 2.4 kV with a pulse duration of 4.3 ms. The transformed cells were plated on LB agar containing kanamycin (50 μg/mL) and incubated at 28 °C for 16 h to obtain single colonies representing the primary homologous recombinants. These primary recombinants were subsequently streaked on LB agar supplemented with 10% (*w*/*v*) sucrose and incubated at 28 °C for 16 h to select single colonies corresponding to the secondary homologous recombinants.

PCR validation of the secondary homologous recombinants was performed by extracting total genomic DNA from selected colonies and amplifying it with verification primers sRNA0024-up-F and sRNA0024-down-R. The 10 μL PCR mixture contained 1.0 μL template DNA, 1.0 μL of each primer (10 μmol/L), 5 μL of 2× Pfu Master Mix (Beijing Lanbo Biotech Co., Ltd., Beijing, China), and nuclease-free water to a final volume of 10 μL. The amplification program consisted of an initial denaturation at 94 °C for 3 min, followed by 28 cycles of 94 °C for 30 s, 60 °C for 30 s, and 72 °C for 90 s. Sanger sequencing of the resulting amplicon spanning the deletion junction confirmed successful deletion of the sRNA0024 locus, and the verified mutant was designated ΔsRNA0024.

### 2.4. Construction of the sRNA0024-Complemented Strain in P. plecoglossicida

The complemented strain C-ΔsRNA0024 of *P. plecoglossicida* was constructed with slight modifications to previously described methods [48]. A 986 bp fragment encompassing the sRNA0024 locus was amplified from the genomic DNA of *P. plecoglossicida* NZBD9 using primers C_sRNA0024-F and C_sRNA0024-R. Both the amplified fragment and the plasmid pCM130/tac were digested with *BsrGI* and *NsiI* (New England Biolabs, Ipswich, MA, USA) and subsequently ligated using T4 DNA ligase (Beijing Baori Yi Biotechnology Co., Ltd., Beijing, China). The ligation mixture was transformed into *Escherichia coli* DH5α competent cells by heat shock, and transformants carrying the recombinant plasmid pCM130/tac-C-sRNA0024 were selected on LB agar containing tetracycline (10 μg/mL). The verified plasmid was then introduced into the ΔsRNA0024 mutant strain of *P. plecoglossicida* by electroporation, and successful complementation was confirmed by PCR using primers sRNA0024-F and sRNA0024-R, as well as by testing for tetracycline resistance.

### 2.5. RT-qPCR Validation of the ΔsRNA0024 and C-ΔsRNA0024strains of P. plecoglossicida

Total RNA was extracted from *P. plecoglossicida* NZBD9, ΔsRNA0024, and C-ΔsRNA0024 strains using a bacterial RNA extraction kit (Beijing Quanshijin Biotechnology Co., Ltd., Beijing, China) according to the manufacturer’s instructions. First-strand cDNA was synthesized from total RNA using TransScript^®^ All-in-One First-Strand cDNA Synthesis SuperMix (TransGen Biotech, Beijing, China). Quantitative real-time PCR (RT-qPCR) was performed with primers RT-sRNA0024-F and RT-sRNA0024-R. The NZBD9 wild-type strain was used as the calibrator, 16S rRNA served as the internal reference, and the relative expression level of sRNA0024 in ΔsRNA0024 and C-ΔsRNA0024 was calculated using the 2^–ΔΔCt^ method. Each 20 μL reaction contained 10 μL of PerfectStart^®^ Green qPCR SuperMix (TransGen Biotech, Beijing, China), 1 μL each of the forward and reverse primers (RT-sRNA0024-F/RT-sRNA0024-R or RT-16S-F/RT-16S-R), 2 μL of cDNA template, and 6 μL of RNase-free water. Thermal cycling conditions were as follows: 94 °C for 30 s, followed by 40 cycles of 94 °C for 5 s and 60 °C for 30 s.

### 2.6. Biological Characterization of P. plecoglossicida NZBD9, ΔsRNA0024, and C-ΔsRNA0024 Strains

#### 2.6.1. Measurement of Bacterial Growth Curves

Single colonies of NZBD9, ΔsRNA0024, and C-ΔsRNA0024 were inoculated into LB broth and cultured overnight at 18 °C with shaking at 220 rpm until the optical density at 600 nm (OD_600_) reached approximately 0.5. The cultures were then adjusted with fresh LB medium to an initial OD_600_ of 0.20 ± 0.01 and further diluted 10^5^-fold. After thorough mixing, 200 μL of each bacterial suspension was transferred into individual wells of a 96-well microtiter plate (eight technical replicates per strain), while 200 μL of sterile LB served as the blank control. The OD_600_ values were measured at 18 °C over a 48 h period using a multimode microplate reader, and bacterial growth curves were plotted accordingly. All experiments were performed in triplicate.

#### 2.6.2. Assessment of Biofilm Formation

Biofilm formation was quantified using the microtiter plate crystal violet assay. Single colonies of NZBD9 and ΔsRNA0024 were inoculated into LB broth and cultured overnight at 18 °C with shaking at 220 rpm until reaching the mid-logarithmic phase (OD_600_ ≈ 0.5). The cultures were adjusted with fresh LB medium to an initial OD_600_ of 0.30 ± 0.01, thoroughly mixed, and dispensed into 96-well microtiter plates (100 μL per well; eight technical replicates per strain). LB medium (100 μL) served as the blank control. The plates were incubated statically at 18 °C for 24 h. The medium was then removed, and the wells were gently rinsed twice with sterile PBS (200 μL per wash) to remove planktonic cells, followed by drying at 60 °C. Biofilms were stained with 0.1% (*w*/*v*) crystal violet (100 μL per well) for 10 min, the excess dye was removed, and the wells were washed twice with 200 μL of sterile PBS and air-dried. The bound dye was solubilized with 200 μL of 33% (*v*/*v*) acetic acid, and absorbance was measured at 600 nm using a microplate reader to quantify biofilm biomass. All experiments were performed in triplicate.

#### 2.6.3. In Vitro Adhesion Assay

(1) Preparation of mucus

Body-surface mucus from *E. coioides* was gently scraped with glass slides. The collected mucus was kept overnight at 4 °C and then centrifuged at 4000× *g* for 30 min at 4 °C. The supernatant was sequentially filter-sterilized through 0.45 μm and 0.22 μm membranes [49] and stored at −80 °C until use.

(2) Adhesion assay

Mucus (20 μL) was evenly spread on glass slides (25 mm × 75 mm) and air-dried overnight. The surface was then fixed with 4% methanol (200 μL) for 30 min. Overnight cultures of NZBD9 and ΔsRNA0024 were adjusted to an OD_600_ of approximately 0.3. After methanol evaporation, 200 μL of each bacterial suspension was applied to the slides and incubated statically at 18 °C for 2 h. The slides were gently rinsed with sterile PBS to remove nonadherent cells and fixed again with 4% methanol (200 μL). Following methanol evaporation, the attached bacteria were stained with 0.1% (*w*/*v*) crystal violet for 3 min, rinsed with sterile PBS to remove excess dye, and air-dried at room temperature. Adherent cells were observed under a light microscope, and 30 random fields were imaged and counted per slide. Three independent biological replicates were performed for each strain, and the entire experiment was repeated three times.

### 2.7. RNA-Seq Analysis of P. plecoglossicida NZBD9 and ΔsRNA0024 Strains

#### 2.7.1. Preparation of Transcriptome Samples

Cells of *P. plecoglossicida* NZBD9 and ΔsRNA0024 cultured for 24 h on LB plates containing 0.5% agar were harvested by centrifugation at 4000× *g* for 10 min at 4 °C for transcriptomic analysis. Three independent biological replicates were prepared for each strain. The collected bacterial pellets were sent to Shanghai Majorbio Bio-Pharm Technology Co., Ltd. (Shanghai, China) for RNA sequencing (RNA-seq).

#### 2.7.2. Read Alignment to the Reference Genome

Clean reads were aligned to the *P. plecoglossicida* reference genome (NCBI assembly accession no. GCF_003391255.1) using the read alignment software Bowtie2 (version 2.5.1; http://bowtie-bio.sourceforge.net/index.shtml (15 June 2025)), which applies the Burrows–Wheeler transform (BWT) algorithm. The resulting mapped reads were retained for downstream analyses.

#### 2.7.3. Gene Expression Quantification and Differential Expression Analysis

Gene expression levels were quantified using RSEM and expressed as TPM (transcripts per million, normalized counts per one million mapped reads). Differential expression analysis between groups was performed with DESeq2, and genes with Benjamini–Hochberg-adjusted *p* values (Padj) < 0.05 and|log_2_(fold change)| ≥ 1 were considered significantly differentially expressed.

#### 2.7.4. GO and KEGG Enrichment Analyses of Differentially Expressed Genes

Functional enrichment analysis of differentially expressed genes (DEGs) between comparison groups was conducted using GOATOOLS (version 1.5.2; https://github.com/tanghaibao/goatools (accessed 15 June 2025)) for Gene Ontology (GO) enrichment and KOBAS (version 3.0; http://bioinfo.org/kobas; (accessed on 15 June 2025)) for KEGG pathway enrichment. Fisher’s exact test was employed to evaluate statistical significance, and GO terms or KEGG pathways with Benjamini–Hochberg-adjusted *p* values (Padj) < 0.05 were considered significantly enriched.

#### 2.7.5. RT-qPCR Validation of RNA-Seq Data

To validate the RNA-seq results, total RNA was extracted from the NZBD9 wild-type and ΔsRNA0024 mutant strains and reverse-transcribed into cDNA. Using the synthesized cDNA as template, RT-qPCR was performed to quantify the transcript levels of the differentially expressed genes *atpG*, *atpB*, *catA*, *pcaD*, *sdhA*, *odhB*, *paaZ*, and *paaK* (full gene names are listed in Supplementary Table S1). The 16S rRNA gene was used as the internal reference, and relative transcript levels were calculated using the 2^−ΔΔCt^ method. Gene-specific primers were designed for RT-qPCR, and their sequences are listed in Supplementary Table S1.

### 2.8. Experimental Infection of E. coioides

#### 2.8.1. Preparation of Bacterial Suspensions

Freshly cultured *P. plecoglossicida* wild-type (NZBD9) and ΔsRNA0024 strains were grown in broth medium as described above to the exponential growth phase. Bacterial cells were then adjusted spectrophotometrically to an optical density at 600 nm (OD_600_) corresponding to approximately 1 × 10^8^ CFU/mL. The cultures were subsequently serially diluted in sterile phosphate-buffered saline (PBS, pH 7.4) to obtain the desired final concentration of 2.5 × 10^5^ CFU/mL for intraperitoneal injection. Because achieving this inoculum required a large dilution factor, the final suspensions contained only a minimal proportion of spent culture medium. The actual bacterial concentrations in the inocula were verified by spread plating on agar plates and counting colony-forming units (CFU) after incubation. The bacterial suspensions were maintained on ice and used within 2 h.

#### 2.8.2. Survival Analysis

Ninety *E. coioides* individuals were randomly divided into three groups (*n* = 30 per group): NZBD9 infection, ΔsRNA0024 infection, and PBS-injected control. Fish in the infection groups were intraperitoneally injected with 0.2 mL of bacterial suspension containing 5 × 10^4^ CFU per fish, while control fish received 0.2 mL of sterile PBS. Mortality was recorded daily for 10 days post-challenge, and necropsies were conducted to examine spleens for infection-associated gross lesions.

#### 2.8.3. Determination of the Median Lethal Dose (LD_50_)

Fish were randomly divided into 16 groups (10 individuals per group) and intraperitoneally injected with 200 μL of the corresponding bacterial suspension, while PBS was used as the blank control. To determine the median lethal dose (LD_50_) of *P. plecoglossicida* NZBD9 and ΔsRNA0024 in *E. coioides*, bacterial suspensions were prepared as 10-fold serial dilutions and administered at five concentrations ranging from 5.0 × 10^6^ to 5.0 × 10^2^ CFU per fish. Mortality was recorded daily for 10 days post-infection (dpi), and the LD_50_ value at 10 dpi was calculated using SPSS version 22.0 (SPSS Inc., Chicago, IL, USA).

#### 2.8.4. Sample Collection

A cohort of 270 *E. coioides* from the same batch was randomly divided into three groups: NZBD9 infection, ΔsRNA0024 infection, and PBS-injected control, following the infection procedures described in Section 2.8.2. At 4 days post-infection (dpi), spleen tissues were collected for histopathological examination, RNA-seq analysis, and gene expression assays. This time point was chosen based on preliminary challenge trials and survival kinetics, which indicated that 4 dpi corresponds to an early acute phase in which typical clinical signs and splenic lesions are evident in NZBD9-infected fish, while most individuals remain alive, allowing robust comparisons between infection groups. The spleen was selected for detailed histopathological and transcriptomic analyses because it is the primary target organ in visceral white spot disease and a central immune organ in teleost fish that orchestrates systemic innate and adaptive immune responses. Samples for histopathology were fixed in tissue fixative, whereas the remaining spleen samples were snap-frozen in liquid nitrogen and stored at −80 °C until use. Three biological replicates were prepared for each group.

#### 2.8.5. Histopathology

Spleen tissues were fixed in 4% paraformaldehyde (PFA) for at least 24 h at room temperature and then rinsed three times with 70% ethanol. Samples were dehydrated through a graded ethanol series (70%, 85%, and 95%; 20 min each), followed by two changes of 100% ethanol (10 min each). The tissues were transferred to a 1:1 mixture of absolute ethanol and xylene for 10 min and subsequently cleared in xylene (two changes, approximately 5 min each; durations adjusted as needed). Paraffin infiltration was performed at 65 °C for at least 1 h, and tissues were then embedded in paraffin wax. Serial sections (7 μm thick) were floated on prewarmed ultrapure water (45 °C) to flatten, mounted on glass slides, and dried overnight at 45 °C. Sections were dewaxed in xylene (two changes, 10 min each) and rehydrated through a graded ethanol series (100% to 70%; 2 min each). Hematoxylin–eosin (H&E) staining was performed according to the manufacturer’s instructions, with staining times adjusted based on tissue characteristics. After staining, slides were dehydrated through 70–100% ethanol, cleared in xylene, and mounted with a neutral resin sealing medium. Histopathological alterations were examined and imaged under a light microscope.

#### 2.8.6. RNA-Seq Analysis of Spleen Samples

##### Preparation of Spleen Transcriptome Samples

For the RNA-seq experiment, all orange-spotted grouper (*Epinephelus coioides*) were obtained from the same commercial hatchery batch and maintained in a single recirculating seawater system under identical environmental conditions during acclimation and throughout the infection trial. Prior to infection, fish were randomly allocated to the PBS control, NZBD9, and ΔsRNA0024 groups by drawing individuals at random from the holding tank and distributing them into the corresponding tanks at equal stocking densities. For host transcriptome analysis, three biological replicates per group were used, with each replicate consisting of spleen tissue from a single fish. At 4 dpi, fish for sampling were randomly selected from each group, and dissections were performed in a randomized order to reduce handling bias. Total RNA extraction and library preparation for all samples were carried out in a single batch by the same operator, using the same lot of reagents and the same protocol, and all libraries were sequenced together on the same platform and run to minimize technical batch effects. The spleen samples were submitted to Shanghai Majorbio Bio-Pharm Technology Co., Ltd. (Shanghai, China) for transcriptome sequencing.

##### Expression Quantification and Differential Expression Analysis

Gene- and transcript-level abundances were estimated using RSEM and expressed as transcripts per million (TPM). Differential expression analysis between groups was performed with DESeq2 based on the raw count matrices. Genes meeting both criteria—a Benjamini–Hochberg false discovery rate (FDR) < 0.05 and an absolute log_2_(fold change) ≥ 1—were defined as significantly differentially expressed.

##### GO and KEGG Enrichment Analyses

Gene Ontology (GO) enrichment analysis was performed using GOATOOLS with Fisher’s exact test. *p*-values were adjusted for multiple comparisons using the Benjamini–Hochberg (BH) method, and GO terms with adjusted *p* (Padj) ≤ 0.05 were considered significantly enriched. Kyoto Encyclopedia of Genes and Genomes (KEGG) pathway enrichment analysis was conducted using KOBAS, also employing Fisher’s exact test followed by BH correction. Pathways with Padj ≤ 0.05 were regarded as significantly enriched among the differentially expressed genes (DEGs).

##### RT-qPCR Validation

Expression levels of spleen genes *c3*, *pck1*, *wnt5a*, *c8a*, *prlra*, *col4a6*, *ocln*, and *mhc2a *(full gene names are listed in Supplementary Table S1) in *E. coioides* were quantified by RT-qPCR. Total RNA was extracted from fish infected with the NZBD9 wild-type or ΔsRNA0024 mutant strain and reverse-transcribed into cDNA. The β-actin gene was used as the internal reference, and relative expression levels were calculated using the 2^−ΔΔCt^ method. Primer sequences used in this study are listed in Supplementary Table S1.

### 2.9. Statistical Analysis and Data Deposition

Experimental data are presented as the mean ± standard deviation (SD). Data normality was evaluated using the Shapiro–Wilk test. One-way analysis of variance (ANOVA) was conducted in GraphPad Prism version 8.0 (GraphPad Software, San Diego, CA, USA), followed by Tukey’s post hoc multiple-comparisons test. Differences were considered statistically significant at *p* < 0.05.

The raw RNA-seq reads for *P. plecoglossicida* NZBD9 and ΔsRNA0024 have been deposited in the NCBI Sequence Read Archive (SRA) under BioProject accession number PRJNA1128240. The spleen RNA-seq data from *E. coioides* infected with NZBD9 or ΔsRNA0024 have been deposited in the NCBI SRA under BioProject accession number PRJNA1161588.

## 3. Results

### 3.1. Prediction of sRNA0024 Targeting luxR in P. plecoglossicida

**Figure 1:**
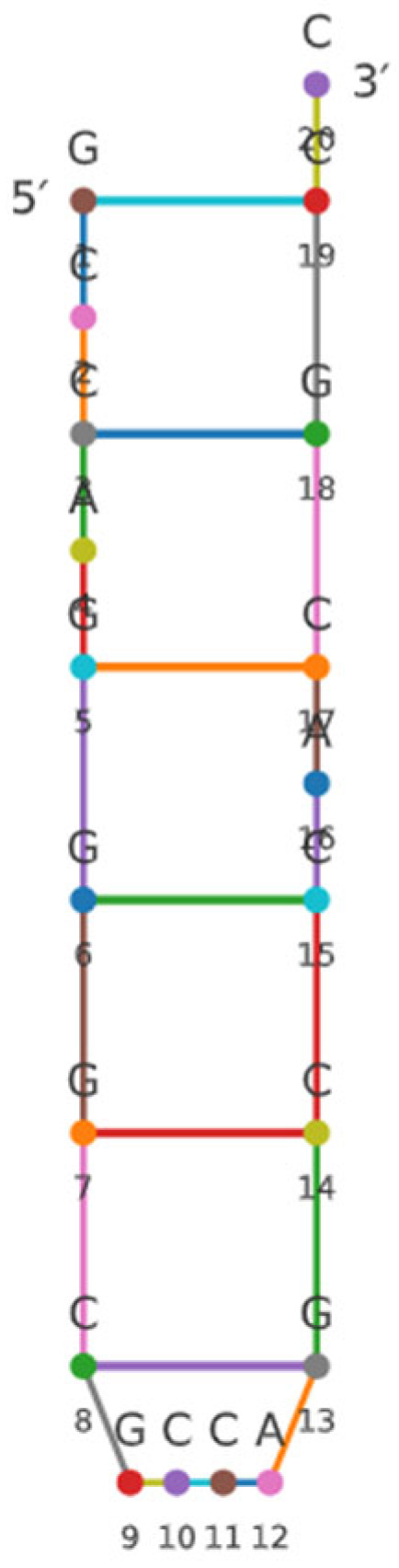
Prediction of sRNA0024 in *P. plecoglossicida.* (Nucleotides are shown as colored circles with letters, with positions 1–20 numbered from the 5′ to 3′ end. Colored lines between circles indicate the predicted base pairs generated by RNAfold).

In silico analysis identified a candidate small RNA, designated sRNA0024 (5′-GCCAGGGCGCCAGCCACGCC-3′). Across multiple target-prediction algorithms (see Section 2.2), sRNA0024 was predicted to base-pair with *luxR* mRNA and to act post-transcriptionally by repressing translation and/or promoting mRNA degradation, rather than influencing transcription initiation. The predicted secondary structure of sRNA0024 is presented in Figure 1.

### 3.2. Verification of ΔsRNA0024 Knockout and C-ΔsRNA0024 Complementation in P. plecoglossicida NZBD9

**Figure 2:**
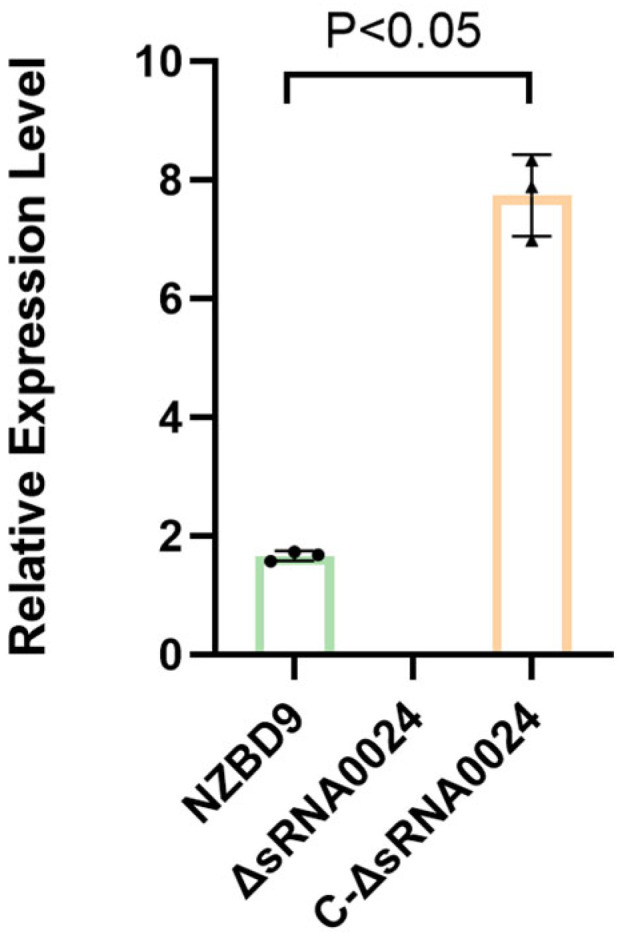
Quantitative RT–PCR analysis of sRNA0024 expression in *P. plecoglossicida* strains. Data are presented as the mean ± standard deviation (SD; *n* = 3).

To verify the successful construction of the ΔsRNA0024 and C-ΔsRNA0024 strains, cDNA synthesized from NZBD9, ΔsRNA0024, and C-ΔsRNA0024 was used as template to assess sRNA0024 transcript levels by RT-qPCR. As shown in Figure 2, an amplicon corresponding to sRNA0024 was detected in the complemented strain (C-ΔsRNA0024), with expression levels exceeding those of the wild-type NZBD9, whereas no amplification was observed in the ΔsRNA0024 knockout. These results confirm the successful generation of both the ΔsRNA0024 deletion mutant and the sRNA0024-complemented strain.

### 3.3. Comparative Growth Analysis of ΔsRNA0024 and C-ΔsRNA0024 Strains

**Figure 3:**
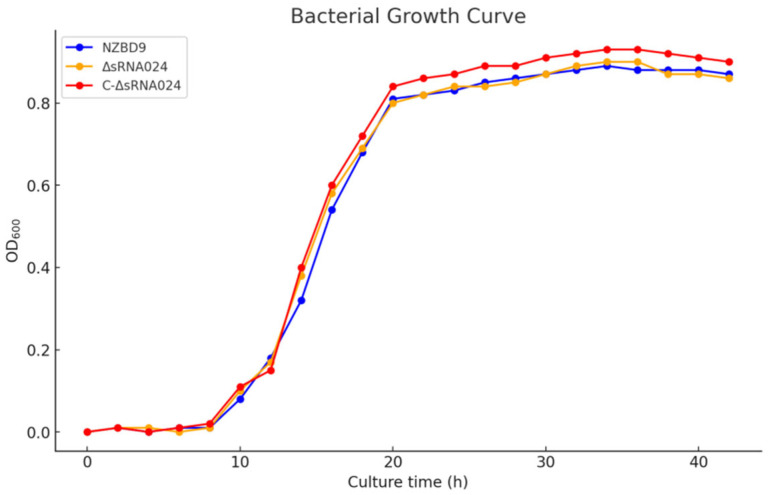
Growth curves of *P. plecoglossicida* NZBD9, ΔsRNA0024, and C-ΔsRNA0024 measured at OD_600_ every hour for 48 h. Data are presented as the mean ± standard deviation (SD; *n* = 8). No significant differences were observed among the strains (*p* > 0.05).

To assess whether the ΔsRNA0024 and C-ΔsRNA0024 strains exhibited any growth defects compared with the wild-type NZBD9 strain, growth curves of all three strains were recorded under identical culture conditions. As shown in Figure 3, no significant differences were observed among the strains throughout the experimental period (*p* > 0.05), indicating that sRNA0024 is not directly involved in bacterial growth.

### 3.4. Biofilm Formation

**Figure 4:**
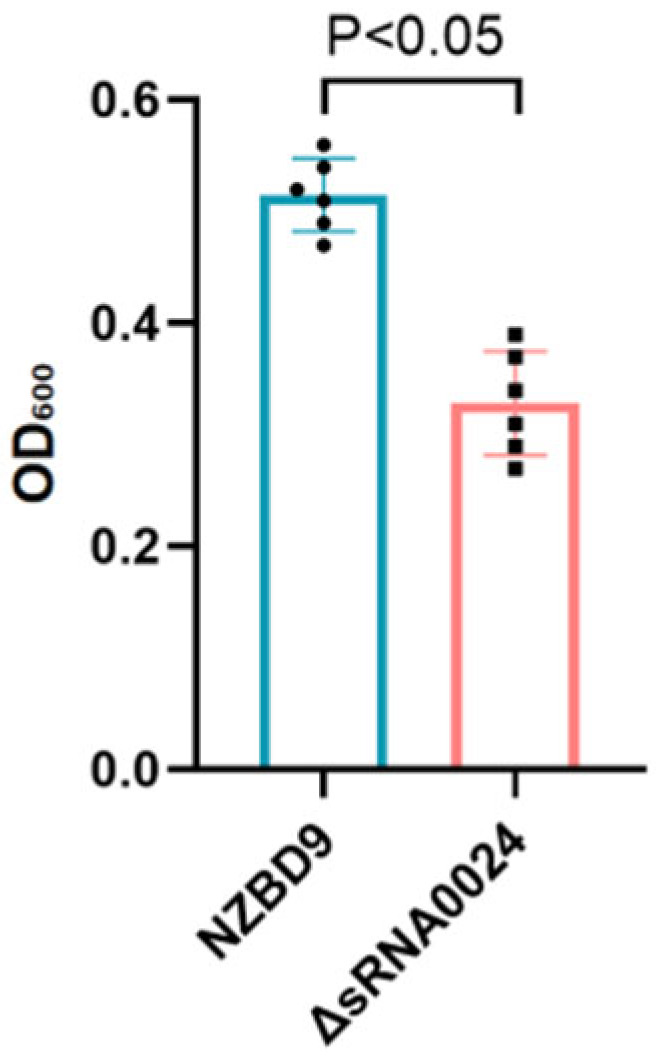
Biofilm formation of the NZBD9, ΔsRNA0024 strains of *P. plecoglossicida*.

Biofilm biomass was quantified using the crystal violet microtiter-plate assay. As shown in Figure 4, after 24 h, the wild-type NZBD9 strain exhibited an OD_600_ value of 0.52 ± 0.04, whereas the ΔsRNA0024 mutant measured 0.34 ± 0.08 (mean ± SD). Statistical analysis indicated that biofilm formation by the ΔsRNA0024 mutant was significantly reduced compared with the wild type (*p* < 0.05; one-way ANOVA followed by Tukey’s test). These results indicate that loss of sRNA0024 markedly diminishes biofilm formation in *P. plecoglossicida*.

### 3.5. In Vitro Adhesion

**Figure 5:**
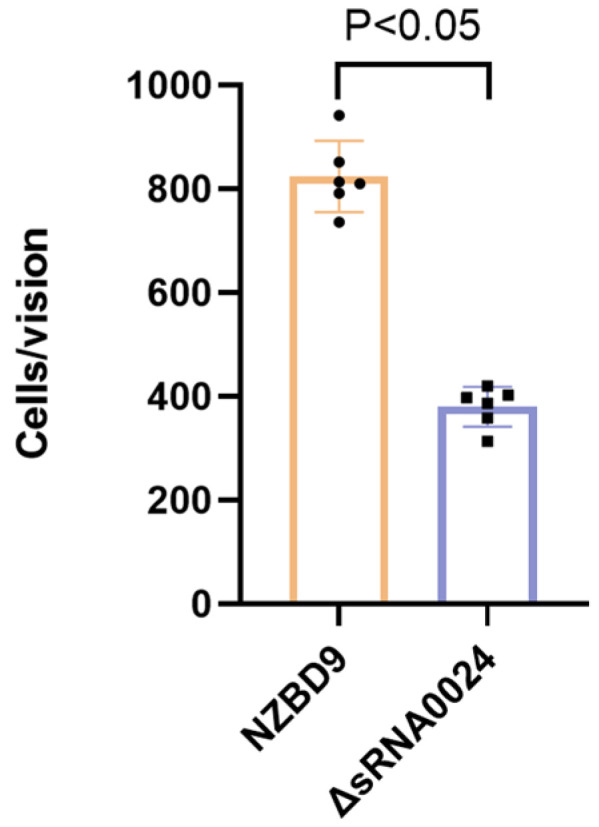
Adhesion ability of the NZBD9, ΔsRNA0024 strains of *P. plecoglossicida*.

Adhesion to *E. coioides* surface mucus was evaluated by crystal violet staining and light microscopy (1000× magnification). As shown in Figure 5, the mean number of adherent bacteria per field was 824 ± 62 for the wild-type NZBD9 strain and 380 ± 34 for the ΔsRNA0024 mutant (mean ± SD). Statistical analysis revealed that adhesion by the ΔsRNA0024 mutant was significantly reduced compared with the wild type (*p* < 0.05; one-way ANOVA followed by Tukey’s post hoc test). These results indicate that loss of sRNA0024 markedly diminishes the ability of *P. plecoglossicida* to adhere to host mucus.

### 3.6. Transcriptomic Analysis of the NZBD9 Wild Type and the ΔsRNA0024 Mutant

#### 3.6.1. Quality Assessment of Bacterial Transcriptome Data

Total RNA was extracted from the NZBD9 and ΔsRNA0024 strains, mRNA was enriched, and RNA-seq libraries were constructed and sequenced on the Illumina HiSeq 4000 platform. In total, 31.32 Gb of clean bases were obtained, with ≥3.1 Gb generated per sample. The proportion of Q30 bases exceeded 96.04% for all libraries. Clean reads were aligned to the *P. plecoglossicida* reference genome, with mapping rates ranging from 96.45% to 97.66%. These quality metrics indicate high sequencing accuracy and consistency, ensuring the reliability of subsequent analyses.

#### 3.6.2. Differential Gene Expression Analysis

**Figure 6:**
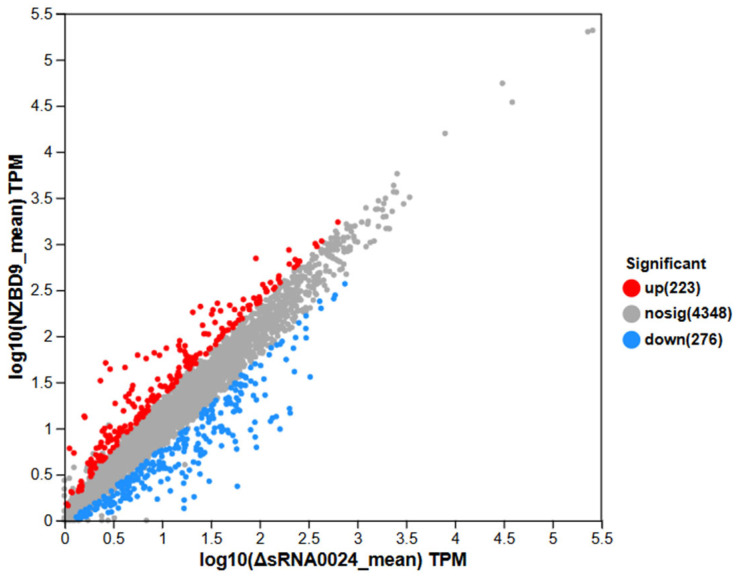
Scatter plot of differential gene expression between NZBD9 and ΔsRNA0024 sample groups: comparison of expression levels and distribution of significantly differentially expressed genes.

Differential expression analysis was conducted using DESeq2 based on normalized read counts to identify genes exhibiting significant expression changes between groups. In the comparison between the NZBD9 and ΔsRNA0024 strains, 223 genes were significantly upregulated and 276 genes were significantly downregulated, while 4348 genes showed no significant difference in expression (Figure 6). Thus, approximately 10% of all detected genes were differentially expressed, indicating a distinct transcriptional reprogramming between the wild-type and mutant strains.

Comparison of mean TPM (transcripts per million) values between the NZBD9 and ΔsRNA0024 groups revealed distinct expression patterns (Figure 6). Highly expressed genes clustered along the upper right quadrant, representing core, constitutively active genes with minimal variation between groups, thus reflecting overall transcriptional stability. In contrast, genes deviating from the diagonal line exhibited pronounced differential expression. Upregulated genes (upper right of the plot) were expressed at markedly higher levels in the wild-type NZBD9 strain, whereas downregulated genes (lower left) were expressed at lower levels relative to the ΔsRNA0024 mutant. These distribution patterns highlight a clear transcriptional divergence associated with sRNA0024 deletion.

#### 3.6.3. Impact of sRNA Knockout on luxR Expression

Differential expression analysis based on high-throughput RNA-seq data (see Supplementary Table S2) revealed a marked downregulation of *luxR* transcription in the ΔsRNA0024 mutant. This result indicates that sRNA0024 positively regulates *luxR* expression at the transcript level, thereby providing molecular evidence that links this sRNA to quorum-sensing signal transduction and its associated regulatory networks.

#### 3.6.4. GO Enrichment Analysis

**Figure 7:**
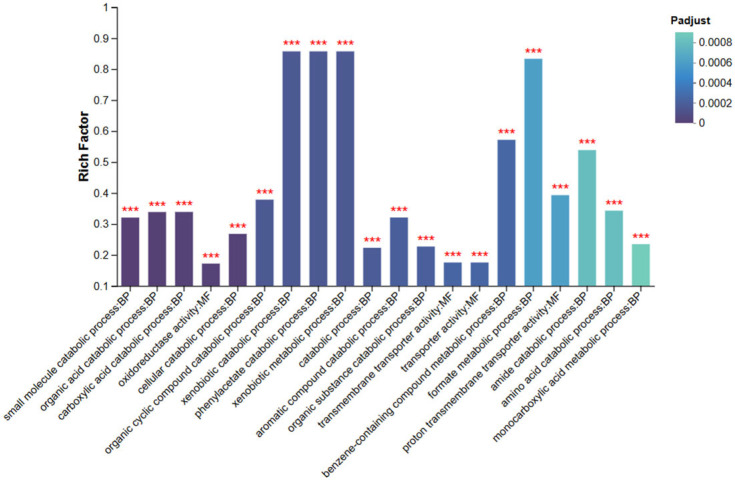
Bar chart of GO enrichment analysis for the NZBD9 and ΔsRNA0024 sample groups. *** indicates significantly enriched GO terms with adjusted *p*-value (Padjust) < 0.001.

Gene Ontology (GO) enrichment analysis was performed using GOATOOLS with Fisher’s exact test to identify the major functional categories of differentially expressed genes (DEGs). GO terms with an adjusted *p* value (Padj < 0.05) were considered significantly enriched. As shown in Figure 7, at both the Biological Process (BP) and Molecular Function (MF) levels, significantly enriched terms included “small molecule catabolic process,” “organic acid catabolic process,” “oxidoreductase activity,” and “transmembrane transporter activity.”

These GO terms indicate that the DEGs are mainly associated with metabolic, transport, and redox processes. The enrichment of small molecule and organic acid catabolic pathways suggests that sRNA0024 deletion affects central metabolic reprogramming in *P. plecoglossicida*, potentially reflecting an adaptive shift in energy utilization. Enrichment of transmembrane transporter activity implies enhanced nutrient or metabolite exchange across the membrane to accommodate altered metabolic demands.

Moreover, enrichment of oxidoreductase activity—closely linked to electron transfer and environmental stress responses—suggests modulation of cellular redox balance and signal transduction pathways. Collectively, these findings demonstrate that sRNA0024 deletion triggers specific metabolic and regulatory adjustments, highlighting the functional importance of these DEGs. GO terms marked with *** denote highly significant enrichment, confirming the robustness of the analysis.

#### 3.6.5. KEGG Pathway Enrichment Analysis

**Figure 8:**
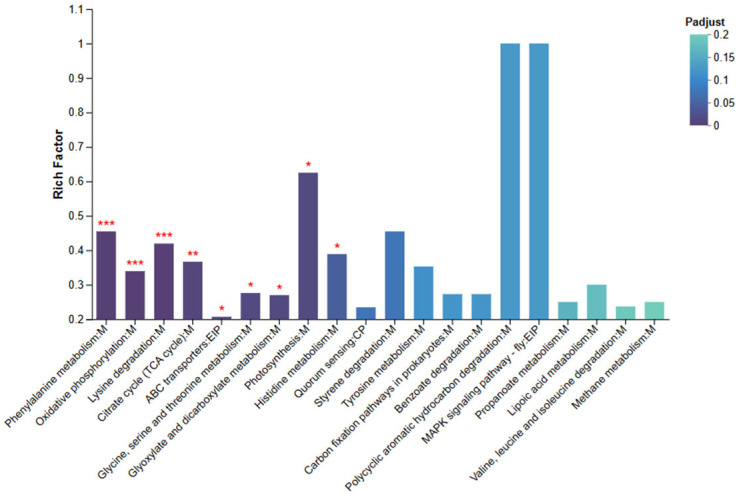
KEGG Enrichment Bar Chart for NZBD9 and ΔsRNA0024 Sample Groups. Asterisks indicate significantly enriched pathways: * Padjust < 0.05, ** Padjust < 0.01, and *** Padjust < 0.001.

KEGG pathway enrichment analysis was conducted using an R script following the same criteria as the GO analysis, with pathways showing adjusted *p* values (Padj< 0.05) considered significantly enriched. As shown in Figure 8, significantly enriched pathways included “Phenylalanine metabolism,” “Oxidative phosphorylation,” “Lysine degradation,” “TCA cycle,” “ABC transporters,” “Histidine metabolism,” and “Photosynthesis.” In addition, moderate enrichment was observed in the “MAPK signaling pathway” and “Benzoate degradation.”

The enrichment of metabolism-related pathways—such as Phenylalanine and Lysine degradation and the TCA cycle—suggests that deletion of sRNA0024 affects central carbon and amino acid metabolism in *P. plecoglossicida*. Enrichment of Oxidative phosphorylation implies altered cellular energy production and redox balance, whereas the ABC transporter pathway points to changes in nutrient uptake and metabolite export. The presence of enriched signaling pathways, including MAPK signaling, further indicates that stress-response and adaptive signaling processes were modulated following sRNA0024 deletion.

Notably, the KEGG “Photosynthesis” pathway was enriched in the bacterial transcriptome. Inspection of the underlying genes indicated that they encode redox and electron-transfer proteins annotated to this pathway based on homology to photosystem components in other bacteria, rather than true photosynthetic machinery in *P. plecoglossicida*.

Collectively, these results demonstrate that the differentially expressed genes are predominantly involved in metabolic reprogramming, energy generation, membrane transport, and signal transduction, highlighting the profound impact of sRNA0024 on the physiological and regulatory network of *P. plecoglossicida*.

#### 3.6.6. qRT-PCR Validation

**Figure 9:**
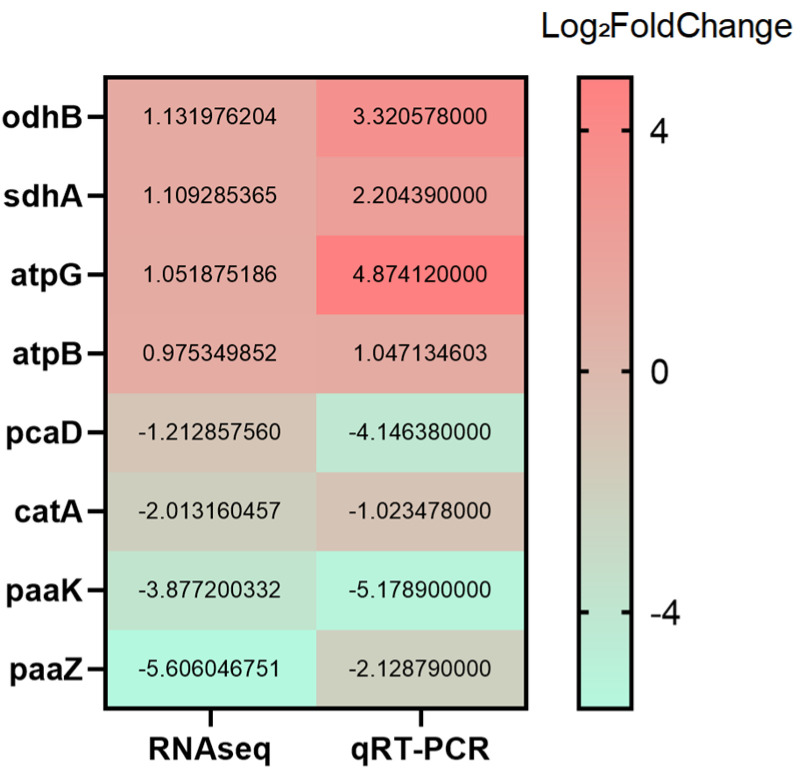
Verification of DEGs by RT-qPCR.

Based on the GO and KEGG enrichment results, representative genes were selected for qRT-PCR validation to confirm the RNA-seq data. Genes associated with the ATP synthase complex (*atpG*, *atpB*), the β-ketoadipate pathway (*catA*, *pcaD*), the tricarboxylic acid (TCA) cycle (*sdhA*, *odhB*), and phenylalanine metabolism (*paaZ*, *paaK*) were analyzed. As shown in Figure 9, although minor differences in fold-change magnitude were observed, the overall expression trends of up- and down-regulation were consistent between the qRT-PCR and RNA-seq datasets, supporting the reliability of the transcriptomic analysis.

### 3.7. Effect of sRNA0024 Deletion on the Virulence of P. plecoglossicida

#### 3.7.1. Comparison of Virulence Between NZBD9 and the ΔsRNA0024 Mutant in the *E. coioides* Infection Model

To evaluate virulence in *E. coioides*, fish were intraperitoneally injected with 10-fold serial dilutions of NZBD9 or the ΔsRNA0024 mutant. Median lethal dose (LD_50_) values were determined from cumulative mortality at 10 days post-infection (dpi). As shown in Table 1, the LD_50_ of the ΔsRNA0024 mutant was approximately 3.83-fold higher than that of the wild-type NZBD9 strain, indicating a substantial attenuation of virulence following sRNA0024 deletion.

#### 3.7.2. Survival Analysis of *E. coioides* Following Infection

**Figure 10:**
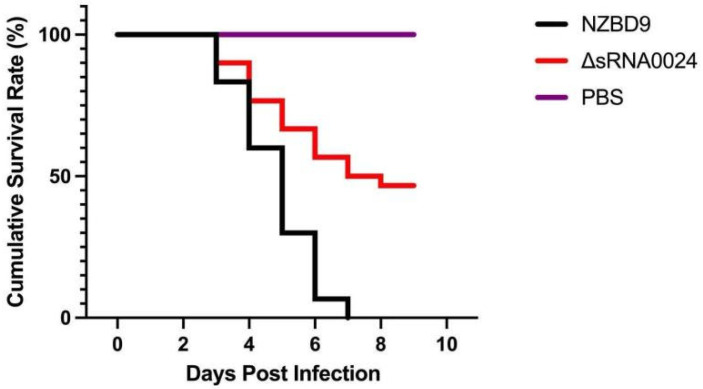
Cumulative survival rate of *E. coioides* infected with the NZBD9, ΔsRNA0024 strains of *P. plecoglossicida*.

To further assess the effect of sRNA0024 deletion on the virulence of *P. plecoglossicida*, 10-day survival was monitored in orange-spotted grouper (*E. coioides*) following intraperitoneal challenge with the wild-type NZBD9 strain, the ΔsRNA0024 mutant, or PBS. As shown in Figure 10, mortality in the NZBD9 group began at 3 days post-infection (dpi), and all fish died by day 8. In contrast, mortality in the ΔsRNA0024-infected group also began at 3 dpi but plateaued thereafter, with a final survival rate of 46%. No deaths occurred in the PBS-injected control group throughout the experimental period.

**Figure 11:**
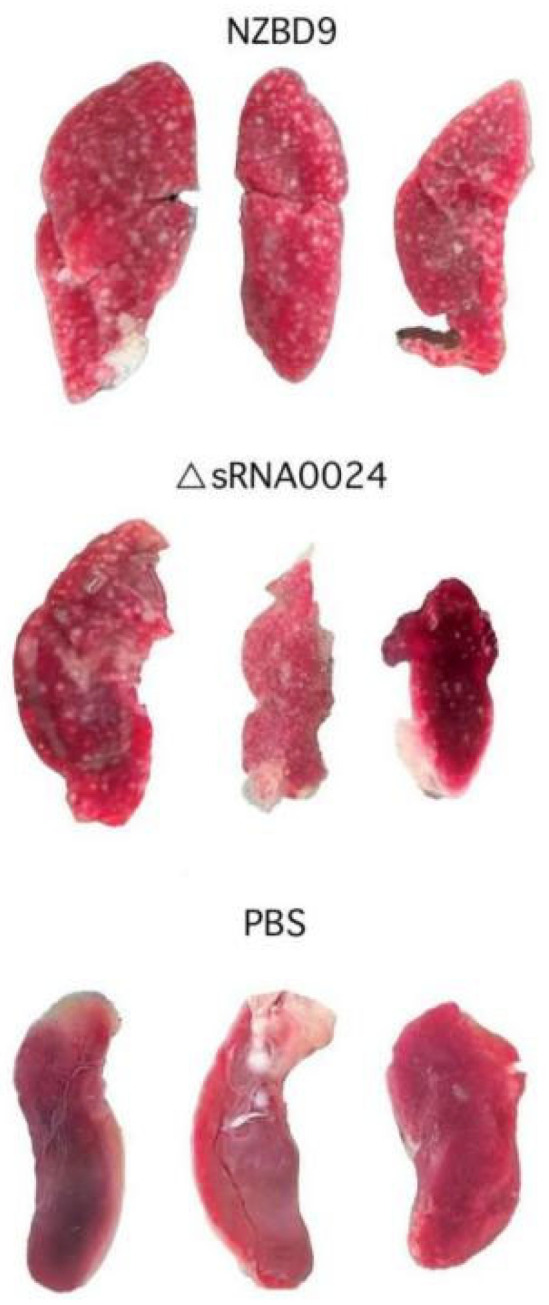
Symptoms of white nodules in the spleen of *E. coioides* infected with the NZBD9, ΔsRNA0024 strains at 4 dpi. All spleens were photographed at the same distance, and each spleen is approximately 2–3 cm in length.

In naturally and experimentally infected fish, the spleen consistently exhibits the earliest and most severe white nodule formation and tissue disruption, and is therefore regarded as the primary target organ in *P. plecoglossicida*-associated visceral white spot disease. Because the teleost spleen is a major secondary lymphoid organ that integrates systemic innate and adaptive immune responses, it represents a key site at which *P. plecoglossicida* interacts with and perturbs host immunity. As shown in Figure 11, at 4 dpi, fish in the NZBD9-infected group displayed clear clinical signs, including lethargy, reduced feed intake, darkened body color, and occasional erratic swimming. Gross examination revealed marked splenomegaly with diffuse congestion and numerous white nodules of varying size scattered on the splenic surface and within the parenchyma. By contrast, fish infected with the ΔsRNA0024 mutant showed only mild lethargy and slight enlargement of the spleen, with few or no visible white nodules, and no overt external abnormalities. The PBS-injected control group remained clinically normal throughout the experiment and showed no gross lesions on necropsy.

#### 3.7.3. Histopathological Examination of Spleen Tissues

**Figure 12:**
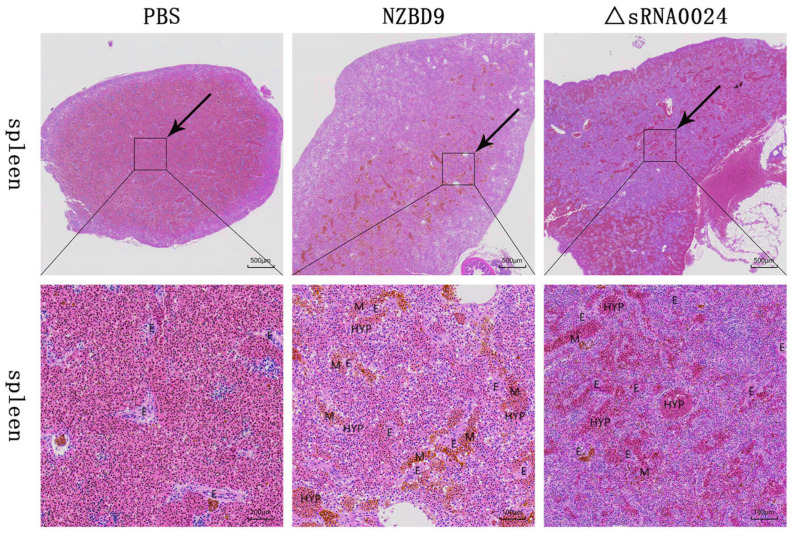
H&E-stained spleen sections of *E. coioides* infected with the NZBD9 and ΔsRNA0024 strains of *P. plecoglossicida* at 4 dpi. Upper panels: low-magnification views (scale bar = 500 μm); lower panels: high-magnification views (scale bar = 100 μm). Note: ellipsoid lumen (E), macrophage (M), hyperemia (HYP).

H&E-stained spleens collected at 4 days post-infection (dpi) from *E. coioides* were evaluated with particular attention to splenic ellipsoids and associated inflammatory changes (Figure 12). In PBS-injected controls, splenic ellipsoids were small and regularly rounded, with narrow lumina and thin perivascular macrophage sheaths. Only a few resident macrophages were present, and the surrounding splenic tissue showed no obvious congestion, hemorrhage, or inflammatory cell accumulation.

In fish infected with the wild-type NZBD9 strain, splenic ellipsoids were strikingly enlarged. Their lumina were markedly dilated and surrounded by thickened cellular sheaths packed with macrophages and other inflammatory cells. Numerous brown–yellow granular deposits within these macrophages were consistent with hemosiderin, and there was prominent inflammatory infiltration and erythrocyte accumulation around the ellipsoids, indicating severe inflammatory congestion.

In the ΔsRNA0024-infected group, the overall morphology of splenic ellipsoids was closer to that of PBS controls. Ellipsoid lumina showed only mild dilation, their perivascular sheaths were relatively thin, and macrophage and inflammatory cell infiltrates were notably reduced compared with the NZBD9 group. However, vascular congestion of splenic vessels and sinusoids remained evident, indicating that inflammatory congestion was still present but much less severe than in wild-type-infected fish.

#### 3.7.4. RNA-Seq Analysis of *E. coioides* Immune Responses to ΔsRNA0024 Infection

##### Quality Assessment of RNA-seq Data

Using a de novo RNA-seq approach, spleen transcriptomes were profiled from *E. coioides* at 4 days post-infection (dpi) with either the wild-type NZBD9 (control) or the ΔsRNA0024 mutant (experimental). In total, 39.74 Gb of clean bases were obtained, with ≥6.23 Gb per sample. The proportion of Q30 bases exceeded 96.07% across all libraries, indicating high sequencing quality suitable for downstream analyses.

##### Assessment of De Novo Assembly

Clean reads from all samples were assembled de novo with Trinity, followed by standard post-assembly optimization. The final assembly contained 55,708 unigenes and 76,660 transcripts, with an N50 length of 2749 bp.

##### Differential Expression Analysis

**Figure 13:**
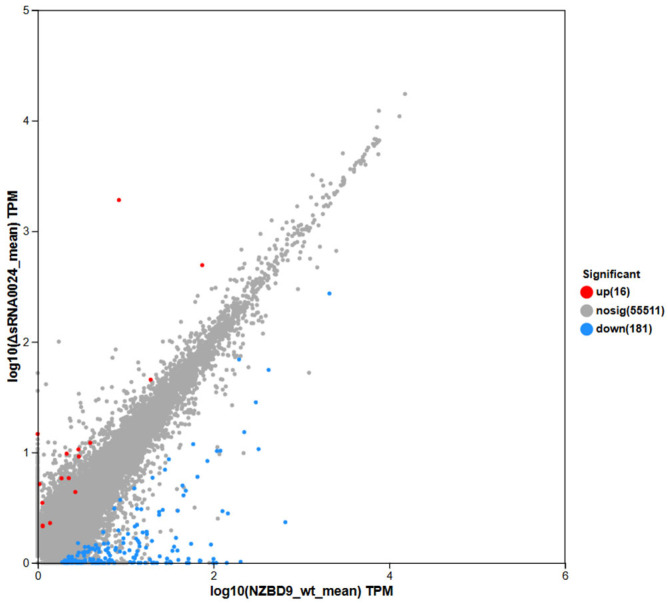
DEGs scatter plot in *E. coioides* spleen: ΔsRNA0024 vs. NZBD9.

Based on expression quantification, differential gene expression between groups was assessed with DESeq2 using thresholds of |log2FC| ≥ 1 and Padj < 0.05. Relative to the NZBD9-infected group, the ΔsRNA0024-infected group exhibited 197 differentially expressed genes (DEGs), including 16 upregulated and 181 downregulated transcripts (Figure 13).

##### GO Enrichment Analysis

**Figure 14:**
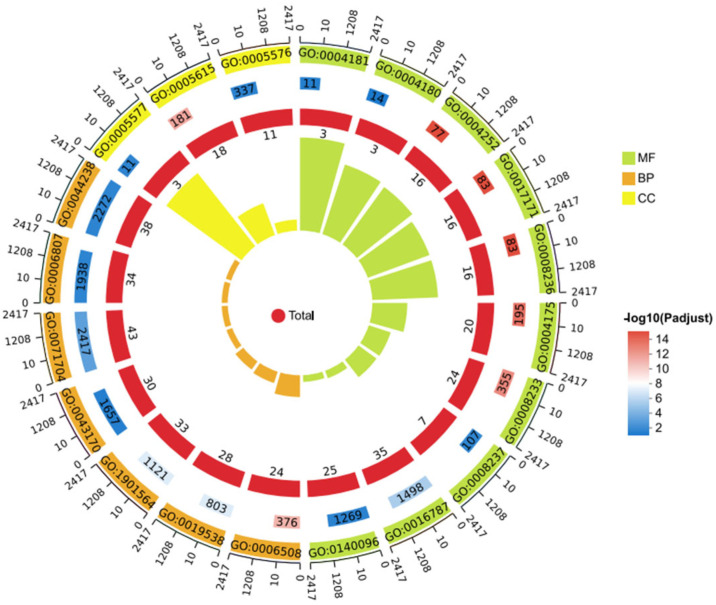
GO enrichment of spleen DEGs in *E. coioides* infected with *P. plecoglossicida* ΔsRNA0024 and wild-type NZBD9.

GO enrichment of DEGs (top 20 terms) revealed the following categories (Figure 14). Biological process (BP): proteolysis; protein metabolic process; organonitrogen compound metabolic process; organic substance metabolic process; primary metabolic process; nitrogen compound metabolic process; macromolecule metabolic process. Cellular component (CC): extracellular space; fibrinogen complex; extracellular region. Molecular function (MF): serine-type endopeptidase activity; serine hydrolase activity; serine-type peptidase activity; endopeptidase activity; peptidase activity; hydrolase activity; metallopeptidase activity; catalytic activity, acting on a protein; metallocarboxypeptidase activity; carboxypeptidase activity.

##### KEGG Pathway Enrichment Analysis

**Figure 15:**
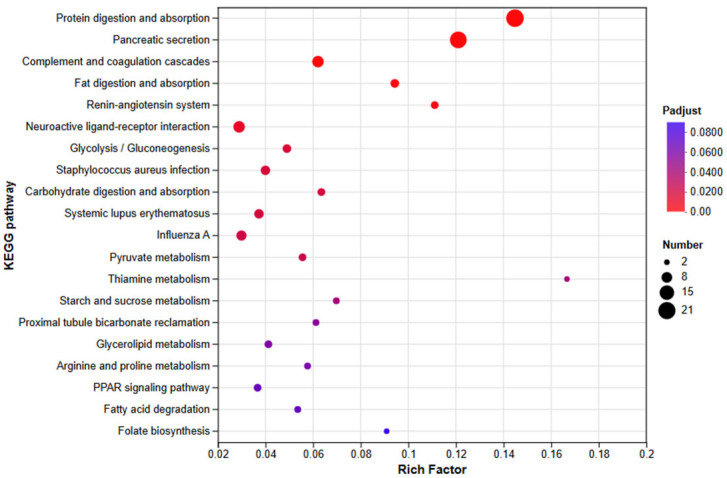
KEGG enrichment analysis of DEGs in *E. coioides* after infection with *P. plecoglossicida* the ΔsRNA0024 and NZBD9 mutant.

All DEGs were subjected to KEGG enrichment (Figure 15). In total, 157 pathways were annotated, of which 54 were significantly enriched (Padj ≤ 0.05); the top 20 are highlighted in Figure 15. Immune-related enrichment spanned three KEGG level-2 categories—immune system, signal transduction, and signaling molecules and interaction. Within the immune system, enriched pathways included complement and coagulation cascades, hematopoietic cell lineage, neutrophil extracellular trap formation, platelet activation, intestinal immune network for IgA production, leukocyte transendothelial migration, antigen processing and presentation, Th1 and Th2 cell differentiation, Th17 cell differentiation, and the chemokine signaling pathway. For signal transduction, enriched pathways comprised AMPK, Hedgehog, PI3K–Akt, FoxO, cAMP, HIF-1, JAK–STAT, mTOR, Hippo, Wnt, Calcium, and cGMP–PKG signaling pathways. Under signaling molecules and interaction, enrichment was observed for neuroactive ligand–receptor interaction, cytokine–cytokine receptor interaction, cell adhesion molecules (CAMs), viral protein interaction with cytokine and cytokine receptor, and ECM–receptor interaction.

##### RT-qPCR Validation of RNA-seq Data

**Figure 16:**
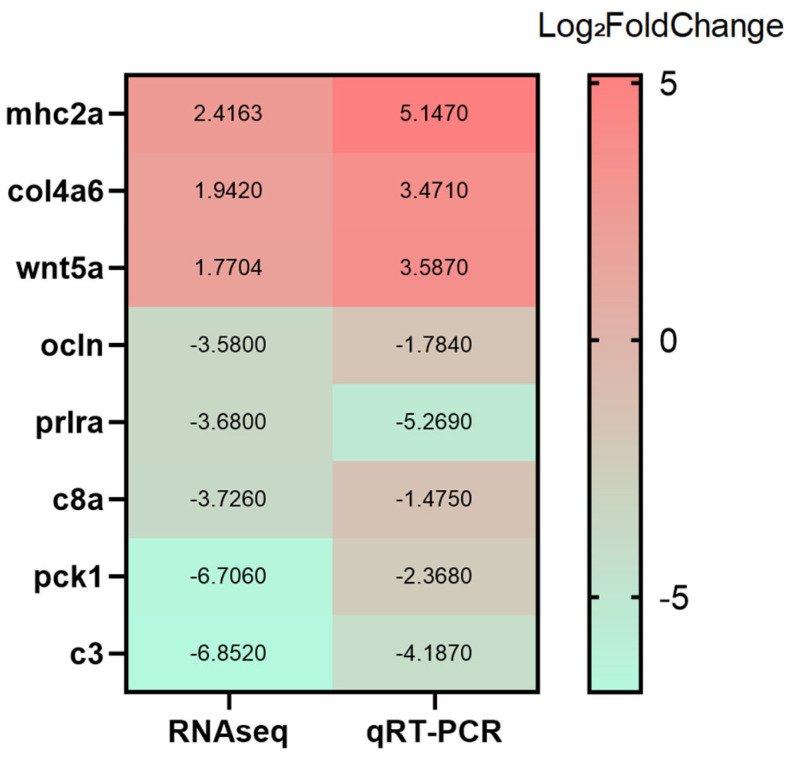
Verification of differentially expressed genes by RT-qPCR.

Based on the transcriptomic analysis, eight representative DEGs were selected for RT-qPCR validation: *c3* (neutrophil extracellular trap formation), *pck1* (FoxO signaling), *wnt5a* (Wnt signaling), *c8a* (complement and coagulation cascades), *prlra* and *col4a6* (PI3K–Akt signaling), and *ocln* and *mhc2a* (cell adhesion molecules). As shown in Figure 16, RT-qPCR-derived expression levels exhibited trends consistent with the RNA-seq results, supporting the accuracy and reliability of the transcriptomic dataset.

## 4. Discussion

In recent years, the roles of small RNAs (sRNAs) in bacterial gene regulation and virulence have garnered considerable attention [50,51,52,53]. To elucidate upstream regulation of *luxR* in *P. plecoglossicida*, we used in silico prediction to identify a candidate sRNA targeting *luxR* mRNA, designated sRNA0024. A deletion mutant (ΔsRNA0024) was constructed by double-crossover homologous recombination, and a complemented strain (C-ΔsRNA0024) was generated in the same background. Using cDNA from NZBD9, ΔsRNA0024, and C-ΔsRNA0024 as templates, we assayed sRNA0024 transcripts by RT-qPCR: specific amplification was detected in NZBD9 and C-ΔsRNA0024 but absent in ΔsRNA0024, confirming successful deletion and complementation. A limitation of this study is that we did not experimentally demonstrate a direct base-pairing interaction between sRNA0024 and *luxR* mRNA; at present, this proposed interaction is inferred only from base-pairing prediction and transcriptomic data, and future studies will employ reporter assays and mutational analyses of the predicted binding site to validate this mechanism.

Growth-curve analysis showed no significant difference in in vitro proliferation between the ΔsRNA0024 mutant and the wild-type NZBD9, whereas biofilm formation and adhesion were both reduced in ΔsRNA0024 relative to the wild type. These phenotypes support a model in which the sRNA0024–*luxR* module maintains the steady-state level of *luxR* mRNA post-transcriptionally—likely via effects on stability and/or translation—thereby influencing LuxR abundance and the LuxR-dependent outputs of biofilm formation, secretion, and virulence. On this basis, the sRNA0024 node may represent an upstream component of the LuxR-family regulatory network and a potential integration point for QS-like signaling. This inference is consistent with observations in *P. plecoglossicida* NB2011, where deletion of the LuxR-solo homolog PplR reduces biofilm formation, providing within-species corroboration [15]. Taken together, our results indicate that loss of sRNA0024, while not altering growth in vitro, down-modulates QS-linked outputs by post-transcriptionally affecting *luxR*/LuxR, in line with the view that QS preferentially governs “costly/public-goods” traits and consistent with the observed attenuation in vivo [54].

Comparative transcriptomics between the NZBD9 wild type and the ΔsRNA0024 mutant revealed DEGs significantly enriched in the tricarboxylic acid (TCA) cycle, oxidative phosphorylation, and amino-acid/aromatic-compound metabolism, alongside enrichment of ABC transporters. This pattern, consistent with the observed repression of *luxR* in the knockout, supports a model in which the sRNA0024–*luxR* module maintains the steady-state level of *luxR* mRNA and/or promotes its translation, thereby sustaining LuxR abundance and coordinately tuning a “metabolism–membrane transport–virulence” network; accordingly, adhesion and biofilm-related phenotypes are attenuated downstream. Our results therefore support a positive role of sRNA0024 in maintaining *luxR* to ensure QS output. Notably, this contrasts with the canonical negative regulation of *luxR*/*hapR* by Qrr sRNAs in *Vibrio* (although Qrr3 can positively activate *aphA*) [55,56,57], suggesting a species-specific sRNA–LuxR regulatory scheme in *P. plecoglossicida*.

Artificial infection assays showed that the LD_50_ of the ΔsRNA0024 mutant was ~3.83-fold higher than that of the wild type, and at an equivalent inoculum the 10-dpi survival rate was 46% (versus 0% for NZBD9), with markedly slower mortality kinetics in the mutant group. Together with the in vitro phenotypes and transcriptomic enrichments (remodeling of the TCA cycle, oxidative phosphorylation, and ABC transporters), these data indicate that sRNA0024 maintains the steady-state *luxR* transcript to sustain quorum-sensing–dependent virulence outputs such as adhesion and biofilm formation. Loss of sRNA0024 reallocates energy metabolism and transmembrane transport, resulting in a pronounced attenuation of in vivo virulence.

Histopathological examination of spleens from hosts infected with the wild type versus the ΔsRNA0024 mutant demonstrated markedly attenuated in vivo virulence of ΔsRNA0024, with reduced tissue injury. These findings further support a model in which sRNA0024 maintains the *luxR* transcript and quorum-sensing (QS) activity to sustain multifactorial virulence traits (adhesion, biofilm). Upon sRNA0024 deletion, virulence is significantly diminished and host histologic damage is mitigated—characterized by less necrosis and pronounced hyperemia.

Comparative spleen transcriptomes from hosts infected with the wild type versus the ΔsRNA0024 mutant yielded 197 DEGs (16 upregulated, 181 downregulated). GO enrichment highlighted terms related to extracellular proteolysis and peptidase activity, the fibrinogen complex, and the extracellular region. KEGG analysis further indicated significant enrichment of innate-immunity and hemostasis pathways—complement and coagulation cascades, platelet activation, neutrophil extracellular trap (NET) formation, leukocyte transendothelial migration, and the chemokine signaling pathway—as well as adaptive-immunity pathways including antigen processing and presentation and Th1/Th2/Th17 cell differentiation. Multiple inflammation–metabolism signaling axes (PI3K–Akt, mTOR, JAK–STAT, HIF-1, AMPK) were also enriched. Given the predominance of downregulated DEGs, these data indicate that, relative to wild-type infection, ΔsRNA0024 elicits a generally blunted host immune–inflammatory and complement–coagulation amplification response, with a downward shift in the inflammatory–metabolic signaling set point. This aligns with histology (less necrotizing damage and pronounced hyperemia) and in vivo attenuation (higher LD_50_, improved survival). Mechanistically, the pattern is consistent with reduced *luxR* transcript and QS activity upon loss of sRNA0024, leading to diminished virulence-effector output and weaker inflammatory triggers.

Host transcriptomic and histopathological evidence indicates that infection with the ΔsRNA0024 mutant suppresses amplification of the coagulation–complement and protease networks, dampens neutrophil recruitment and neutrophil extracellular trap (NET) formation, and lowers the burden on inflammation–metabolism signaling axes (PI3K–Akt, JAK–STAT, HIF-1/mTOR, AMPK), accompanied by contraction of ECM/adhesion and cytokine–receptor interaction modules. Overall, the response shifts from the broad hyperactivation observed with the wild type to a lower-amplitude immune reaction with mitigated tissue damage. This causal chain—QS attenuation → reduced colonization/secretion/extracellular enzyme output → avoidance of overactivation of immune cascades—is consistent with the concept of quorum sensing as a virulence amplifier [58,59].

Most bacterial sRNAs regulate target-gene expression by masking or exposing the ribosome-binding site (RBS), altering 5′-UTR secondary structure, or recruiting/occluding ribonucleases [60,61]. In our study, the *luxR* transcript was downregulated in the ΔsRNA0024 mutant and restored by complementation, supporting a model in which sRNA0024 positively regulates *luxR* post-transcriptionally—potentially via Hfq-mediated base pairing that stabilizes *luxR* mRNA and/or enhances its translation [62]. Accordingly, the sRNA0024–*luxR* module constitutes an upstream node in quorum-sensing (QS) control. By maintaining high *luxR* transcript levels, sRNA0024 sustains QS-dependent virulence traits, including adhesion, biofilm formation, and chemotaxis. Deletion of sRNA0024 triggers broad rewiring of metabolism and transmembrane transport, mitigates host tissue injury (less necrosis and pronounced hyperemia), and results in attenuated virulence in vivo, as evidenced by an increased LD_50_ and improved host survival.

From a prevention and ecological-application standpoint, *Pseudomonas plecoglossicida* can trigger outbreaks of visceral white spot disease in large yellow croaker (*Larimichthys crocea*), indicating a high outbreak risk within the environment–host–pathogen triad. Our data suggest that the sRNA0024–*luxR* axis may function as an upstream regulatory node that couples metabolism, quorum sensing (QS), and virulence—on the one hand promoting in vivo colonization and tissue injury, and on the other providing an actionable anti-virulence target. In practical terms, this axis could be exploited by developing sequence-specific antisense oligonucleotides or RNA mimics that interfere with sRNA0024 or its interaction with *luxR* mRNA, or by screening small molecules that destabilize sRNA0024 or inhibit *luxR* activity, thereby dampening QS-associated virulence without directly killing the bacteria. In addition, ΔsRNA0024 or *luxR*-attenuated strains with stable loss of virulence could be further evaluated as candidates for live-attenuated vaccines or for competitive exclusion in farming systems. Implementing such anti-virulence strategies, in combination with optimized husbandry practices (elevated dissolved oxygen, rational stocking density and feeding, improved water circulation and microbiota management), could attenuate pathogenicity while reducing selection for antimicrobial resistance, thereby alleviating host hypoxia/coagulation and inflammatory stress and interrupting the “metabolism–QS–virulence–tissue injury” amplification loop. Taken together, our evidence suggests that the post-transcriptional sRNA0024–*luxR* circuitry may represent an upstream control node in *P. plecoglossicida* virulence, with potential as an anti-virulence intervention target and a translatable, eco-friendly route for precision control of aquaculture diseases.

## 5. Conclusions

In this study, we show that the small RNA sRNA0024 is a key determinant of *Pseudomonas plecoglossicida* virulence in orange-spotted grouper (*Epinephelus coioides*). Deletion of sRNA0024 reduced biofilm formation, adhesion, and in vivo pathogenicity, and alleviated splenic immunopathology. Transcriptomic analyses further indicated that sRNA0024 modulates *luxR*-associated quorum-sensing virulence networks, highlighting the sRNA0024–*luxR* module as a potential antivirulence target for controlling visceral white spot disease in marine aquaculture.

## Figures and Tables

**Table 1:** Median lethal dose (LD_50_) values of *P. plecoglossicida* NZBD9 and ΔsRNA0024 strains in *E. coioides*.

Strain	Bacterial Injected Dose (CFU/Fish)	The Number of Sample	The Number of Death Samples	LD_50_ (CFU/Fish)
NZBD9	5.0 × 10^6^	10	10	2.81 × 10^3^
	5.0 × 10^5^	10	10	
	5.0 × 10^4^	10	9	
	5.0 × 10^3^	10	6	
	5.0 × 10^2^	10	2	
ΔsRNA0024	5.0 × 10^6^	10	10	1.08 × 10^4^
	5.0 × 10^5^	10	9	
	5.0 × 10^4^	10	7	
	5.0 × 10^3^	10	4	
	5.0 × 10^2^	10	0	
Control	PBS	10	0	—

## Data Availability

All figures and tables used to support the results of this study have been included.

## Supplementary Materials

The following supporting information can be downloaded at https://www.mdpi.com/article/10.3390/ani15243623/s1, The following supporting information is available: Table S1: Primers used for PCR and qRT-PCR; Table S2: Detailed differential expression data used in this study.
