# Supplementary material for: Deletion of sRNA0024 Reduces Virulence of Pseudomonas plecoglossicida and Alleviates Host Immune Injury in Epinephelus coioides
Source: Animals (Basel). 2025 Dec 17;15(24):3623. doi: 10.3390/ani15243623 (PMC12729329; doi:10.3390/ani15243623)
Supplement: Supplementary file 1 [file animals-15-03623-s001.zip › Supplementary_Table_S1_Primers_for_PCR_and_qRT-PCR.pdf]

Supplementary Table S1: The sequence of Primers for PCR and qRT-PCR

**gene name**	Base sequence (From 5' to 3')
gyrB	F: 5'-TGCTGAAGGACGAGCGTTCG-3'<br>R: 5'-ATCATCTTGCCGACAACAGC-3'
luxR	F: 5'- GCGGAAACGGATAATGGCTCA -3'<br>R: 5'-GCGGACAAGTTTGGGAAATAGGT-3'
16S rDNA	F: 5'-TTCATCGCCACTGCACCTG-3'<br>R: 5'- GTTCTTGCCCTTCTCCCAC-3'
**pK18*mobsacB***	Ext-F: 5'-CTAGCGTTGACTGCTACGAT-3′<br>Int-R: 5'-GCTGATGTTGTCGTCGTTGA-3'
β-actin	F: 5'-GGCTACTCCTTCACCACCACA-3'<br>R: 5'-GGGCAACGGAACCTCTCAT-3'
sRNA0024	F: 5′-TGGCGCCAGCTTCGCCTTTG-3′<br>R: 5′-GCGCTCCCGTCGCGCGGTGC-3′
atpB**（ATP synthase subunit beta, mitochondrial）**	F: 5'- ATGGCAGCAGAAACCGCTTC-3'<br>R: 5'- TTTAACCATTCTTCGTGAGC-3'
atpG**（ATP synthase subunit gamma, mitochondrial）**	F: 5'- GAGATCTCCTGGGTGATCGA-3'<br>R: 5'- CGTTACGTGGAGTCGCAGGT-3'
catA**（Catechol 1,2-dioxygenase）**	F: 5'- CGTCTGCAACTGGAAGTCGA-3'<br>R: 5'- TGTGGGATGACTTTGCCTAC-3'
odhB**（2-oxoglutarate dehydrogenase E2 component）**	F: 5'- ATGGCTATCGAGATCAAAGC-3'<br>R: 5'- TCCAGGACGACCTTGTCGGT-3'
paaK**（Phenylacetate－CoA ligase）**	F: 5'- GATGCCGATGTACGTCTTGA-3'<br>R: 5'- CTTTATGAGATTCATCTGTA-3'
paaZ**（Phenylacetic acid degradation protein PaaZ）**	F: 5'- ATGTCTGACGCCCCAACCCT-3'<br>R: 5'- ACGGCTTCGGCGAAGTCCGG-3'
pcaD**（3-oxoadipate enol-lactonase）**	F: 5'-CTGCATGAAGCGGCCATGCT -3'<br>R: 5'-GCGCATCTGCCAGATGCTCG-3'
sdhA**（Succinate dehydrogenase flavoprotein subunit）**	F: 5'-GGTCACAAGACAGCCGTGGT -3'<br>R: 5'-CCCTTGACGGTATCGTACAT-3'
C3**（Complement C3）**	F: 5'- ATGGAGCCTGTAGAGAGGGA-3'<br>R: 5'- GTGCATCCCTGATATCAGAGA-3'

pck1 **（Phosphoenolpyruvate carboxykinase 1 (GTP), cytosolic）**	F: 5'- CAGAAGATGGTGAACCCTGC-3'<br>R: 5'- CTCCGAAGATGATGGCCTCG-3'
wnt5a **（Wnt family member 5A）**	F: 5'-TTCACTTATGCCATCAGCGC -3'<br>R: 5'-CAGTCTCGCGGAAGGTCTTT-3'
c8a **（Complement component 8 alpha chain）**	F: 5'-GTGGTGTCTCAGTTGGGATC -3'<br>R: 5'-AATGAACCCAAGGTCCTGGC-3'
prlra **（Prolactin receptor a）**	F: 5'-ACACCCGCTCTGGTTGGATC -3'<br>R: 5'-GAGATGCACCTTGCAGGCTG-3'
col4a6 **（Collagen type IV alpha 6 chain）**	F: 5'-CGGGAAGAACGAGTCTCCTC -3'<br>R: 5'-AGACGATGTCAGCCACGGTG-3'
ocln **（Tight junction protein occludin）**	F: 5'-CTACCAGACCAGCTACTCAC-3'<br>R: 5'-CCTCTCTTCTTCTAGATCTG-3'
mhc2a **（Major histocompatibility complex class II alpha chain）**	F: 5'-CTTCAATACTGGCTCAGAGG -3'<br>R: 5'-AGAGCTCAGTACAACGTAGG-3'
